# Progressive Dystrophic Pathology in Diaphragm and Impairment of Cardiac Function in *FKRP* P448L Mutant Mice

**DOI:** 10.1371/journal.pone.0164187

**Published:** 2016-10-06

**Authors:** Anthony Blaeser, Hiroyuki Awano, Bo Wu, Qi-Long Lu

**Affiliations:** McColl-Lockwood Laboratory for Muscular Dystrophy Research, Cannon Research Center, Carolinas Medical Center, 1000 Blythe Blvd., Charlotte, NC 28203, United States of America; University of Minnesota Medical Center, UNITED STATES

## Abstract

Mutations in the gene for fukutin-related protein represent a subset of muscular dystrophies known as dystroglycanopathies characterized by loss of functionally-glycosylated-alpha-dystroglycan and a wide range of dystrophic phenotypes. Mice generated by our lab containing the *P448L* mutation in the *fukutin-related protein* gene demonstrate the dystrophic phenotype similar to that of LGMD2I. Here we examined the morphology of the heart and diaphragm, focusing on pathology of diaphragm and cardiac function of the mutant mice for up to 12 months. Both diaphragm and heart lack clear expression of functionally-glycosylated-alpha-dystroglycan throughout the observed period. The diaphragm undergoes progressive deterioration in histology with increasing amount of centranucleation and inflammation. Large areas of mononuclear cell infiltration and fibrosis of up to 60% of tissue area were detected as early as 6 months of age. Despite a less severe morphology with only patches of mononuclear cell infiltration and fibrosis of ~5% by 12 months of age in the heart, cardiac function is clearly affected. High frequency ultrasound reveals a smaller heart size up to 10 months of age. There are significant increases in myocardial thickness and decrease in cardiac output through 12 months. Dysfunction in the heart represents a key marker for evaluating experimental therapies aimed at cardiac muscle.

## Introduction

Dystroglycanopathies are a heterogeneous group of muscle disorders associated with the aberrant glycosylation of alpha-dystroglycan (α-DG). Alpha-DG is the post-translationally cleaved subunit of the DG polypeptide and a critical component of the dystrophin-glycoprotein complex (DGC) [1,2]. Alpha-DG, through its extensively *N*- and *O*-linked glycosylated glycans, acts as a cellular receptor for laminin and other extracellular matrix (ECM) proteins, including agrin, perlecan, neurexin and pikachurin. Its C terminal is non-convalently linked to a transmembrane partner, β-DG, through which to cytoskeleton proteins. This linkage is crucial for the maintenance of muscle membrane stability [3–5]. In dystroglycanopathies, α-DG is abnormally glycosylated, lacking specific O-Mannosyl glycosylated epitopes considered to be functionally critical for laminin binding (functional glycosylation). The absence of functional glycosylation of α-DG, defined mainly by specific antibodies and binding affinity to laminin, destabilizes binding of the DGC to the ECM and alters membrane permeability, leading to contraction related fiber damage and degeneration. The lack of functional glycan epitopes on α-DG has been linked to at least 18 known or putative glycosyltransferase genes. These include LARGE, *FKRP*, *fukutin*, POMT1/2, POMGnT, ISPD, GTDC2, β2GALNT2, SGK196, β3GNT1, ISPD, TMEM5, DOLK, GMPPB, DMP1, DMP2, and DMP3 [6–17]. The role some individual genes play in the glycosylation of α-DG is well defined. The POMT genes have been identified as key components involved in the synthesis of *O*-mannosyl linked oligosaccharides with a complex of POMT1 and POMT2 providing full *O*-mannosyltransferase activity and POMTGnT1 acting as an *O*-mannose-β-1,2-N-acetylglucosaminyltransferase [7,18,19]. It has also been shown that LARGE is the glycosyltransferase involved in the addition of repeating units of [-3-xylose-α1,3-glucuronic acid-β1-][20–23]. The function of Fukutin and *FKRP* has now been elucidated. Recent publication by Kanagawa et al. has identified both fukutin and FKRP as ribitol 5-phosphate (Rbo5P) transferases [24]. The clinical severity of the dystroglycanopathies associated with these genes varies from mild limb girdle muscular dystrophy with primarily myopathic phenotypes to more severe disorders such as Walker-Warburg syndrome (WWS) and muscle-eye-brain (MEB) disease with prominent central nerve system (CNS) involvement.

*FKRP* mutations are the most common causes of the dystroglycanopathies. Almost all of the *FKRP* mutations are missense point mutations with the C826A mutation being the most common. While no specific correlation between mutation site and disease phenotype has been established, the C826A homozygotes are largely associated with mild limb girdle muscular dystrophy (LGMD) 2I. However, the age of onset of LGMD2I varies considerably between 0.5 to 27 years old and 61% of patients have dystrophic phenotypes before the age of 5 (www.neuromuscular.wustl.edu)[25]. Heterozygous C826A in combination with other mutations presents mainly as LGMD2I, but also associated with more severe forms, including congenital muscular dystrophy (CMD), WWS and MEB [25](biobase-international website). Individuals with the same mutations even within a family can present diseases with significant variation in severity. Factors responsible for such variation remain largely unclear although mutation site is undoubtedly important. LGMD2I affects primarily skeletal muscles with progressive muscle degeneration and loss of function [26]. Lack of sufficient regeneration in human leads to a gradual loss of muscle mass and increase in infiltration with eventual fibrosis and fat deposition.

Dystroglycanopathies affect cardiac muscle variably. Cardiomyopathies are prominent in severe cases of dystroglycanopathies such as CMD and MEB disease, but also prevalent in LGMD2I patients[27–30] with up to 60% frequency [31]. The reported myocardial functional abnormality includes reduced left ventricular ejection fraction, visualized by cardiovascular magnetic resonance imaging (CMR), cardiac conduction defects, mitral regurgitation and dilated cardiomyopathy. Fatty deposition and fibrosis in the septum and inferior wall has also been identified by CMR analysis. Despite the individual description of defects in cardiac physiology and functions, little is understood how the cardiomyopathy progresses histologically and functionally [25].

We have recently generated several mouse models representing *FKRP* mutations in patients and demonstrating a wide range of disease phenotypes as observed in clinics. One mutant mouse strain containing the P448L (C1343T) mutation exhibits the lack of expression of functionally glycosylated α-DG (F-α-DG) in all skeletal muscles except in a small proportion of fibers, termed revertant fibers. This mouse has clear dystrophic pathology in all skeletal muscle from as early as weaning and the disease progresses with age. There is no clear defect in CNS and eyes. This strain of mice has near normal breeding capacity and lives up to more than one year, although they begin to die not long after. The mice therefore represent LGMD2I in clinic and are valuable for studying the disease mechanism(s) [32,33].

Here we evaluated the *FKRP*-*P448Lneo-* (hitherto referred to as *P448Lneo-*) mutant mouse during a 12-month life span with focus on the cardiac muscle and diaphragm for disease progression. Specifically, we monitored cardiac function by echocardiogram at 5 different stages from the disease onset. Our results showed a clear progression of the dystrophic phenotype associated with aging. An impairment of cardiac function as well as severe dystrophic pathology in the diaphragm can be seen by 6 months with further deterioration as the animal ages. These data are consistent with the observation in patients with *FKRP* mutation in clinics and provide us with guidance for using the models to test experimental therapies for their potential on cardiac muscle.

## Materials and Methods

### Animals and ethical statement

*P448Lneo-* mice generated by the McColl Lockwood lab and C57BL/6 (Jackson Laboratory, Bar Harbor, ME, USA) were used. *P448Lneo-* mice contain homozygous missense mutation (c.1343C>T, p.Pro448Leu) in the *FKRP* gene with the Neo^r^ cassette removed from the insertion site. These animals were further back-crossed with C57BL/6 mice. *P448Lneo-* homozygotes showed muscle weakness demonstrated by limb muscle retraction, elevated serum CK and ALT levels, and dystrophic pathology associated with LGMD without clear defect in CNS [32,33].

The study was carried out in strict accordance with the recommendation in the Guide for the Care and Use of Laboratory Animals of the National Institutes of Health. The protocol was approved by the Committee on the Ethics of Animal Experiment of Institutional Animal Care and Use Committee (IACUC) Carolinas Medical Center (01-13-04A). All treatment was performed under isoflurane anesthesia, and every effort was made to minimize suffering. Animals were monitored daily by vivarium staff. Animals were euthanized by isofluorane and cervical dislocation. No animals died prior to experimental endpoint. Animals were housed on a 12h light and dark cycle.

### Histology and immunohistochemistry

Heart and diaphragm muscles were snap-frozen in isopentane chilled in liquid nitrogen. Cross sections of 6μm thickness were cut and stained with hematoxylin and eosin (H&E) and Masson’s Trichrome (MT). Immunohistochemical staining of IIH6 was performed on cross sections of varying age muscle. Frozen sections were dried at room temperature for 15 minutes and subsequently fixed in ice cold Ethanol:Acetic Acid (1:1) for 1 minute. Slides were washed with PBS and blocked with 8%BSA/PBS for 30 minutes at room temperature. IIH6C4 antibody (Millipore, #05–593, Lot# 2200–926) was diluted 1:500 in 1%BSA/1xPBS and added to muscle sections and incubated overnight at 4°C. Negative controls received 1%BSA/1xPBS only. Sections were washed and secondary AlexaFluor 488 goat anti-mouse IgM (A21042, Life Technologies, Carlsbad, CA) was added with 1:500 dilution in PBS and incubated for 3 hours at room temperature. Sections were washed with PBS and mounted with medium containing DAPI (4’,6’-diamidino-2-phenylindole) for nuclear staining. Immunofluorescence was visualized using an Olympus BX51 fluorescent microscopy (Opelco, Dulles, VA, USA). Images were captured using an Olympus DP70 CCD camera system (Opelco, Dulles). For determination of percentage of central nucleated fibers, three random 20X magnification images per section per animal were used. Diaphragm width was determined by measuring the width at six randomly selected areas along the diaphragm length in three random 4x magnification images per section per animal. Percent of fibrosis was determined using ImageJ software to calculate the amount of fibrosis represented by blue coloring in the MT sections (http://rsb.info.nih.gov).

### Protein extraction and western blot

Total protein was extracted from heart and diaphragm muscles using TX-100 buffer (1% Triton X-100, 50mM Tris pH8.0, 150mM NaCl, 0.1% SDS) supplemented with protease inhibitor cocktail (Roche, Germany). Samples were homogenized in TX-100 buffer and the supernatants were collected by centrifugation at 16,000*g* for 10 minutes at 4°C. Protein concentration was determined by Bradford assay (Bio-Rad DC protein assay). Following homogenization 20μg of lysate was loaded on a 4–20% Tris-glycine gel (Invitrogen, Carlsbad, CA, USA). Proteins were transferred to polyvinylidene difluoride (PVDF) membranes with constant ampere of 200mA for 2 hours at 4°C. PVDF membranes were incubated for 1 hour in protein-free T20 blocking buffer (Pierce, Rockford, IL). Primary antibody, IIH6C4, against α-DG was diluted 1:2000 in blocking buffer, added to the membrane and incubated overnight at 4°C. Secondary HRP-Goat anti-mouse IgM (Invitrogen) antibody recognizing the primary antibody was diluted 1:4000 in 20mM Tris pH7.4, 150mM NaCl, 0.1% Tween20. Membranes were incubated for 1 hour at room temperature with secondary antibody. ECL (PerkinElmer, Waltham, MA, USA) was added to the membrane and exposed and processed by a LAS-4000 imaging system (Fujifilm, Valhalla, NY, USA).

### Echocardiogram

Animals anesthetized with 1–4% isoflurane were placed on a mouse monitor pad with nose cone supplying 1–2% isoflurane and oxygen. Electrode gel was applied to the paws which were taped down over the electrocardiogram pads on the monitor platform. Animal heart rate and body temperature was monitored throughout the procedure using the output from the mouse monitor platform. Hair was removed from the chest of the mouse and ultrasound gel was added. Data was gathered using the Bioscan SonixTablet Ultrasound System (Analogic Ultrasound, Peabody, MA, USA). The heart was imaged under M and B mode with the B mode placement imaging the heart at the level of the aortic sphincter. Ejection fraction (EF) was calculated using the equation EF (%) = ((EDV-ESV)/EDV)x100. Stroke volume (SV) was calculated as SV = EDV-ESV. Cardiac output (CO) was calculated as CO = SV x HR. Cardiac index (CI) was calculated as CI = CO/Weight (g). Eliptical representation of heart shape was determined using the equation for elliptical area as area = πAB, where A equals the height radius and B equals the length radius.

### Statistical analysis

For data analysis of echocardiogram student t-test was used. A two-tailed p-value of less than 0.05 was considered statistically significant.

## Results

### Histopathology of diaphragm in different age groups

**Fig 1:**
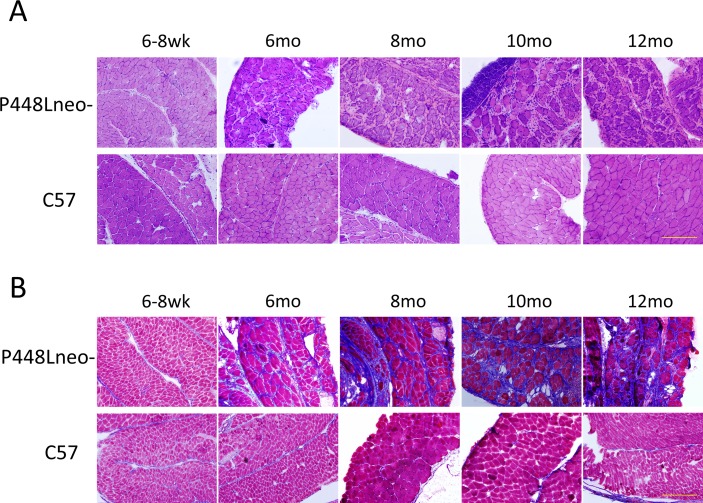
Histology of *P448Lneo-* and *C57* diaphragm at the ages of 6–8 weeks, 6 months, 8 months, 10 months and 12 months. (A) H&E staining. Of note is the increasing amount of extracellular matrix and infiltration of mononuclear cells at 6 months of age in the *P448Lneo-* mutant mice. (B) Masson’s trichrome staining. Blue staining indicates increasing amount of collagen connective tissue. Significant collagen connective tissue replacement can be seen at 6 months of age in *P448Lneo-* mutant diaphragm and increasing through 12 months of age. Little to no blue staining is present in *C57* diaphragm up through 12 months of age. Representative of a minimum of 3 mice per age group. All samples shown at 20X magnification. Yellow bar equal to 200μm.

**Fig 2:**
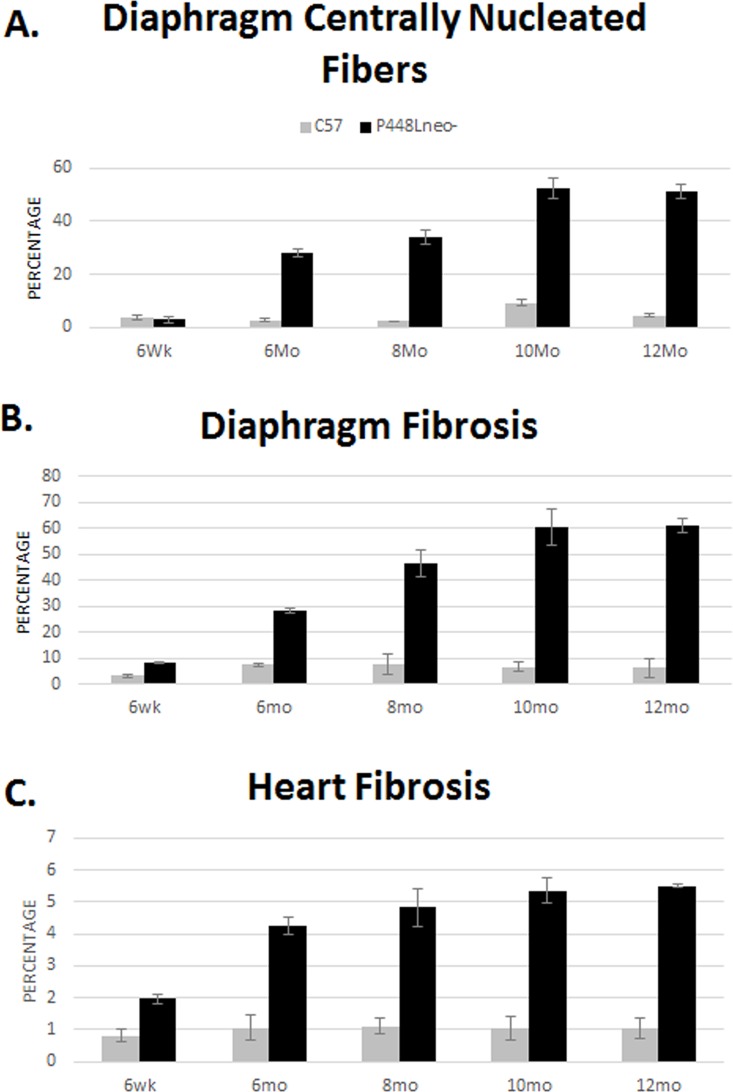
Percentage of central nucleation and fibrosis of diaphragm and heart. (A) Diaphragm central nucleation. Control samples show a slight increase in percentage of centrally nucleated fibers, up to 5%. There is a large increase in centrally nucleated fibers of *P448Lneo-* animals at 6 months of age and reaching approximately 60% by 8 months. (B) Diaphragm fibrosis. Control samples maintain less than 10% fibrosis throughout the 12 month period. *P448Lneo-* samples show a marked increase in fibrosis at 6 months and reaching 60% by 10 months. (C) Heart fibrosis. Control samples maintain approximately 1% fibrosis throughout the 12 month period however *P448Lneo-* samples show a marked increase at around 6 months of age. Heart fibrosis area does not increase more than 6%. Averages are taken using data collected from 3 separate images from 3 animals for each age point (total of 9 images). Error bars represent mean ± SEM.

Our early studies have revealed that the dystrophic phenotype of *P448Lneo-* mutant mice becomes evident as early as 4 weeks after birth [32,33]. The severity of the myopathy in the diaphragm steadily progressed as the animal aged. At 6 weeks of age, the thickness of the diaphragm was similar to that in normal controls with more than 10 layers of even-sized fibers packed closely to each other (Fig 1A). Less than 3% of fibers were centrally nucleated and only sporadically distributed small areas of mononuclear cell infiltration were observed (Fig 2A). Similarly, trichrome staining for collagen did not show a clear difference between the mutant mice and the *C57* wild type mouse, with only about 5% of the tissue stained positive for collagen (Fig 1B). However, by 6 months inflammatory infiltration has spread to the entire organ with greatly reduced fiber density. Large areas of infiltrates, up to ~130 um in diameter and lacking any muscle fiber, were frequently detected. Degenerating fibers containing immunoglobulins were easily detected and centrally nucleated fibers increased to approximately 28%. The remaining fibers appeared highly irregular in shape as well as highly variable in size (Fig 1A). However, cross section thickness of the diaphragm remained similar to the wild type diaphragm with a width of approximately 400 μm. Masson’s Trichrome staining showed a significant increase in the area of collagen of up to 45% replacement at 8 months (Figs 1B and 2B).

By 10 months of age, the area of fibrotic tissue within the diaphragm further increased to approximately 60% (Fig 2) and muscle fibers became isles of variable sizes surrounded by thick bands of fibrotic tissue as shown with the trichrome staining (Fig 1B). The majority of fibers became centrally nucleated. By 12 months, the number of fibers continues to decrease and the cross section thickness decrease significantly, with less than half of the normal diaphragm (~430 μm in *C57* and ~190 μm in *P448L*) and less than 5 lays of fibers in cross section of almost entire diaphragm (Fig 1A).

### Functional glycosylation of α-DG in diaphragm and in cardiac muscle

**Fig 3:**
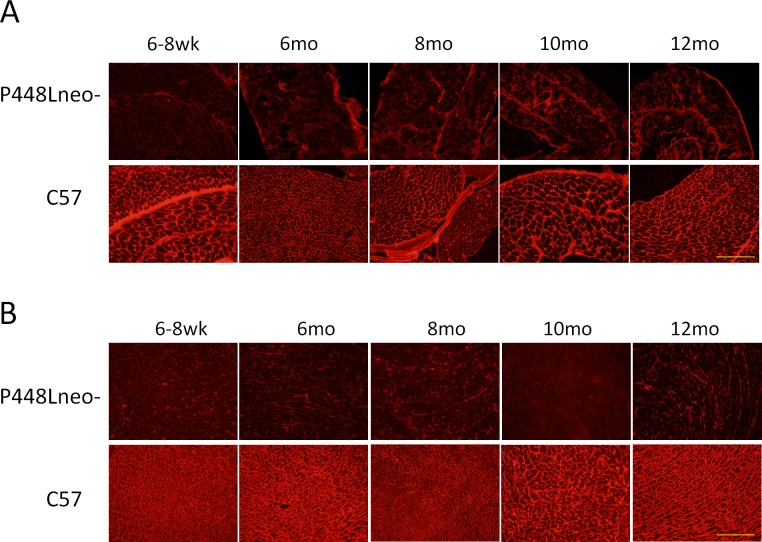
Immunohistochemistry with IIH6C4 antibody for the detection of glycosylated α-DG in diaphragm and heart muscle of *P448Lneo-* mutant and *C57/Bl6* normal control. (A) Diaphragm. Control samples show expression of glycosylated α-DG in muscle fibers throughout a 12 month period. There is an increasing level of background staining, and almost all fibers lackingclear membrane signal for glycosylated α-DG in *P448Lneo-* diaphragm. (B) Heart. Control samples show expression of glycosylated α-DG in muscle fibers throughout a 12 month period. *P448Lneo-* cardiac muscle shows a complete lack of expression of glcyosylated α-DG. Representative of a minimum of 3 mice per age group. All samples shown at 20X magnification. Yellow bar equal to 200μm.

Diaphragm of the *P448Lneo-* mutant mice demonstrated a nearly complete lack of membrane-localized glycosylated α-DG in the majority of muscle fibers detected with IIH6 antibody by immunohistochemistry (Fig 3A).This was observed at all ages from 6 weeks to 12 months. A few fibers with strong membrane staining (revertant fibers) were clearly detected in mice 6 months or younger as described earlier (33). Immunostaining with monoclonal antibody in combination with secondary anti-mouse Igs produced a progressively stronger background staining within the ECM and some fibers in the P448Lneo- mutant mice with age, but this was not observed in the control *C57* mice. The increased intercellular and cytoplasmic staining remained without the primary antibody, thus representing the non-specific binding of mouse intercellular proteins and the specific binding of leaked endogenous Igs by the anti-mouse secondary antibody and was therefore an indicator of increased degeneration of the muscle fibers and damage of vasculature. The strong background staining made the identification of revertant fibers impossible.

Similar to the diaphragm, heart muscle of the P448Lneo- mutant mice showed a complete lack of glycosylated α-DG by immunohistochemistry (Fig 3B) and only one or a few fibers (~5 fibers) were occasionally detected in some mice (1 in 5 mice). However, unlike the diaphragm, background staining remained negligible suggesting a limited degree of degeneration. This is consistent with the histological analysis demonstrating minimal pathology in the heart.

The lack of F-α-DG in both diaphragm and heart was confirmed by western blot at any age group when compared to high level signals in *C57* control tissue (S1 Fig).

### Histopathology of cardiac muscle in different age groups

**Fig 4:**
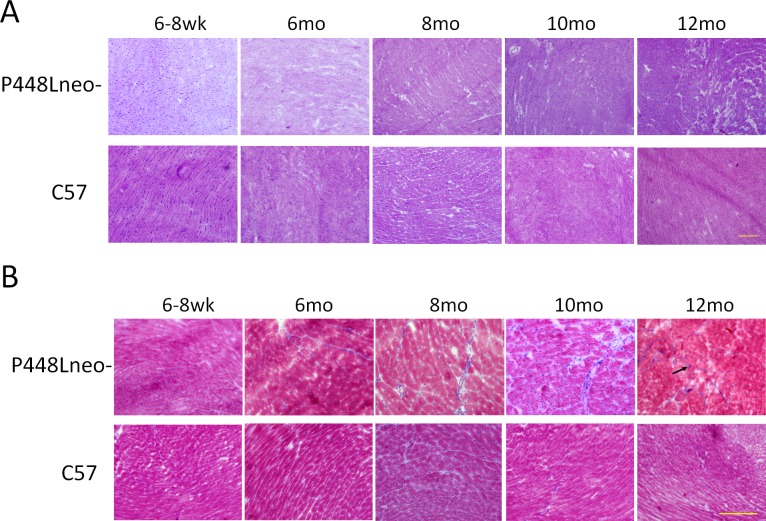
Histology of *P448Lneo-* and *C57* heart at 6–8 weeks, 6 months, 8 months, 10 months and 12 months. (A) H&E staining. Of note is the slight increase in areas containing mononuclear cell infiltration beginning at 10 months of age in *P448Lneo-* mutant heart. The number and size of areas of mononuclear cell infiltration increases by 12 months of age. However the overall amount of affected area in the heart remains limited. (B) Masson’s trichrome staining. Blue staining indicates increasing amount of collagen connective tissue. Areas of *P448Lneo-* mutant heart begin to show increase of connective tissue beginning at 6 months of age up to 12 months of age. No clear replacement is seen in hearts of control *C57* mice up to 12 months of age. Black arrows denote streaks of fibrosis. Representative of a minimum of 3 mice per age group. Samples in A are shown at 10X magnifcation while samples in B are at 20X magnification. Yellow bar equal to 200μm for both.

H&E staining of cardiac muscle from 6 week old *P448Lneo-* mutant mice revealed no obvious infiltration and muscle degeneration. However, some narrow streak of space without muscle fibers were observed in the *FKRP* mutant heart whereas such spaces were not obvious in the wild type cardiac muscle (Fig 4A). These spaces contained few nuclei, but were stained blue with the Mason trichrome thus indicating an increase in ECM component (referred to as fibrotic space). By 6 month of age, the fibrotic space increased in size with an increase in the number of mononuclear infiltrates. This space occupied about 4% of *FKRP* mutant mouse heart tissue compared to 1% in *C57* (Figs 4B and 2C). Sporadic, small areas of mononuclear cell infiltration were also detected. However, degenerating fibers remained undetectable. At 10 months of age, both the fibrotic areas and their size (about 5%) increased further with focal areas of fibrotic tissue clearly identified (Figs 4B and 2C). By 12 month of age fibrotic areas and their size continued to increase reaching approximately 6% (Figs 4B and 2C) of the mutant heart tissue with an increase in areas of mononuclear cell infiltration (Fig 4A).

### Echocardiogram

**Fig 5:**
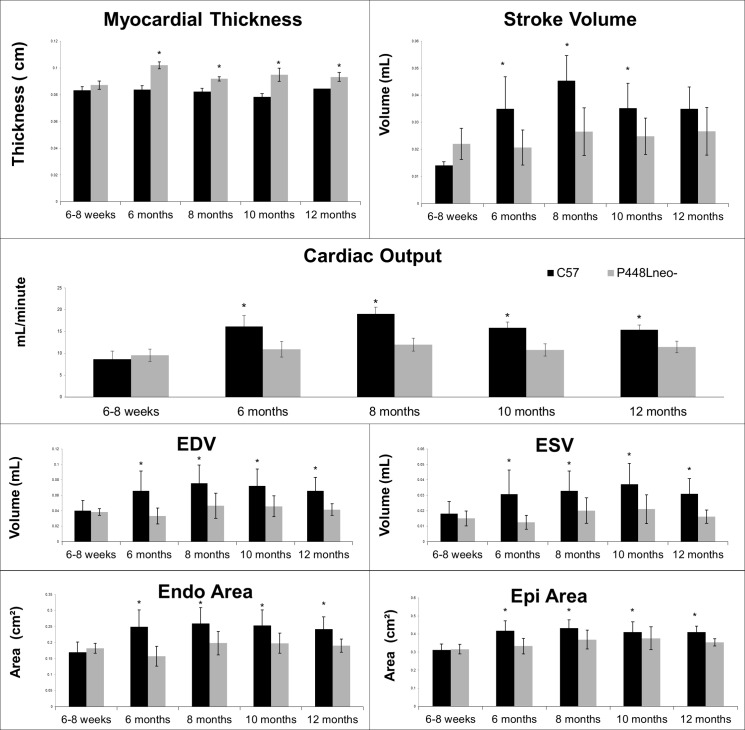
Cardiac function markers in *C57* and *P448Lneo-* mutant mice at 6–8 weeks, 6 months, 8 months, 10 months and 12 months. Values for myocardial thickness, stroke volume, cardiac output, end diastolic volume (EDV), end systolic volume (ESV), endocardial area, epicardial (epi) area, weight and cardiac index (CI) for C57 and P448Lneo- mutant mice were taken at 6–8 weeks, 6 months, 8 months, 10 months (minimum n = 15), and 12 months (minimum n = 6). Statistically significant differences (p<0.05) were observed starting at 8 months for myocardial thickness and 6 months for cardiac output. Stoke volume became statistically significant at 6 months however no significant difference was seen at 12 months. Statistically significant differences (p<0.05) were observed in EDV, ESV as well as endocardial and epicardial area starting at 6 months and remaining until 12 months of age. A larger body weight was observed in C57 mice throughout the 12 month period with a reduced CI starting at 6 months of age and remaining until 12 months. Error bars represent mean ± SEM.

The body weight of the *P448Lneo-* mutant mice at the 6 weeks of age was similar to the age matched *C57* (between 18 and 20g). However, the diastolic length (DL) and systolic length (SL) of the left ventricle of the heart were significantly smaller than those of wild type *C57* (Table 1). Consistently, end diastolic volume (EDV) and end systolic volume (ESV) of the mutant mice were significantly less than those of the *C57*. The difference became even greater by 6 months of age. However, the DL and SL reached maximum in the *C57* by 6 months. In contrast, DL and SL continued to increase in the *P448Lneo-* mutant mice up to 12 month of age, reaching similar length (SL) to and even longer (DL) than the *C57* (Table 1, Fig 5). Despite the smaller size in SL and DL at 6 weeks of age, the myocardial thickness of the *FKRP* mutant mice was greater than that of *C57* mice. The thickness reached the maximum at 6 month of age, and significantly higher than the age matched *C57*, followed by a gradual decline, but remaining higher than the *C57* by the age of 12 months. This was associated with a smaller endocardial area from 6 weeks up to 12 months compared to C57. Taken together (Fig 5) these measurements suggest that P448Lneo- mutant heart experiences structural adaptation with cardiac muscle hypertrophy. This hypertrophy leads to a reduced endocardial area and volume, but also enables the mutant mice to maintain a similar EF throughout the age from 6 weeks to 12 months (Table 1). However, a reduced cardiac output (CO) was noted beginning at 6 months and again lasted up to 12 months of age when compared to the *C57* wild type mice (Fig 5). This reduction in output is therefore likely a result of the reduced ventricle size (Table 1). By the age of 12 months however, the EDV and ESV decreases in both *C57* and mutant mice. This results in no change in the SV (stroke volume) of the *C57* and only a slight increase in the *P448Lneo-* mutant mouse. The overall SV was significantly lower in the mutant mouse than that in the *C57* controls from 6 months onwards despite an increase from 10 to 12 months in the mutant mouse (Table 1). The CO at 12 months was consistent with the SV showing a slight increase from 10 to 12 months in *P448Lneo-* mice while remaining lower than that in *C57*. Due to the reduced weight of the *P448Lneo-* mouse compared to *C57* controls over a 12 month period, the cardiac index (CI) was calculated to account for the effect of a larger weight on the CO. The CI of mutant mice at 6–8 weeks of age was higher than *C57* controls however it becomes lower at 6 months of age with the largest difference at 8 months. However, this difference is reduced by 10 months of age and the CI remains lower in 12 month mutant mice (Fig 5).

**Table 1:** Comparison of cardiac function markers in *C57* (C) and *P448Lneo-* (P) mutant mice at 6–8 weeks, 6 months, 8 months, 10 months and 12 months.

	6wk		6mo		8mo		10mo		12mo	
	P	C	P	C	P	C	P	C	P	C
**Dias Length (cm)**	0.478±0.04	0.595±0.05	0.618±0.07	0.780±0.05	0.668±0.1	0.765±0.07	***(0*.*691±0*.*05)***	***(0*.*722±0*.*07)***	***(0*.*713±0*.*05)***	***(0*.*751±0*.*05***
**Sys Length (cm)**	0.369±0.04	0.494±0.06	0.501±0.08	0.689±0.08	0.558±0.1	0.649±0.06	0.562±0.06	0.620±0.06	***(0*.*609±0*.*06)***	***(0*.*603±0*.*06)***
**EDV (A2C) (mL)**	***(0*.*02±0*.*005)***	***(0*.*04±0*.*01)***	0.033±0.01	0.66±0.03	0.047±0.02	0.076±0.02	0.046±0.01	0.072±0.02	0.041±0.0006	0.066±0.008
**ESV (A2C) (mL)**	***(0*.*006±0*.*002)***	***(0*.*018±0*.*008)***	0.013±0.004	0.031±0.02	0.020±0.008	0.033±0.01	0.021±0.009	0.037±0.01	0.016±0.0003	0.031±0.004
**Endo Area (cm**^**2**^**)**	***(0*.*112±0*.*02)***	***(0*.*17±0*.*03)***	0.158±0.03	0.25±0.05	0.198±0.04	0.259±0.05	0.198±0.03	0.253±0.05	0.190±0.02	0.242±0.04
**Epi Area (cm**^**2**^**)**	***(0*.*239±0*.*02)***	***(0*.*312±0*.*03)***	0.333±0.04	0.418±0.06	0.369±0.05	0.432±0.05	0.376±0.06	0.411±0.06	0.355±0.02	0.41±0.03
**Endo Length (cm)**	0.502±0.04	0.609±0.05	0.636±0.07	0.787±0.05	0.682±0.1	0.768±0.07	***(0*.*704±0*.*05)***	***(0*.*728±0*.*07)***	***(0*.*713±0*.*04)***	***(0*.*75±0*.*05)***
**EF (%)**	71.7±3.6	55.6±6.4	54.5±4.9	62.3±7.1	***(57*.*5±6*.*7)***	***(56*.*9±8*.*8)***	***(49*.*2±4*.*6)***	***(54*.*9±9*.*1)***	***(52*.*0±1*.*7)***	***(53*.*5±0*.*4)***
**SV (mL)**	0.014±0.001	0.022±0.006	0.021±0.006	0.035±0.01	0.027±0.009	0.45±0.009	0.025±0.007	0.035±0.009	***(0*.*027±0*.*009)***	***(0*.*035±0*.*008)***
**Myo-thickness (cm)**	***(0*.*087±0*.*003)***	***(0*.*083±0*.*003)***	0.102±0.003	0.084±0.003	0.092±0.002	0.062±0.003	0.095±0.005	0.078±0.002	***(0*.*093±0*.*003)***	***(0*.*084±0*.*004)***
**CO (mL/min)**	9.59±1.4	8.68±1.9	10.95±1.8	16.22±2.5	12±1.5	19.03±1.6	10.8±1.4	15.8±1.4	11.27±1.4	15.73±1.1
**Weight**	17.5	21.5	33.23	40.89	35.36	43.9	37.67	46.45	37.25	48.25
**CI (CO/Weight)**	0.54	0.40	0.33	0.40	0.34	0.43	0.29	0.34	0.30	0.33

At 6 weeks of age significant differences were demonstrated in the length of the heart as well as the ejection fraction and stroke volume. However, by 6 months of age significant differences are noted among all of the cardiac function markers. The ejection fraction (EF) of the *P448Lneo-* mice begins to normalize at 8 months of age with the length of the heart nearing normal at 10 months. By 12 months of age the systolic (ESV) and diastolic (EDV) volumes as well as the endocardial and epicardial area remain significantly different. Cardiac output (CO) remains significantly lower beginning at 6 months of age through 12 months. The weight of the P448Lneo- mouse remains smaller throughout the 12 month period. The cardiac index (CI) is higher in the 6 week old *P448Lneo-* have a higher cardiac index however it becomes smaller by 6 months of age and remains lower than the *C57* control throughout the 12 month time period. Statistical significance determined through students t-test (p<0.05). Non-statistically significant difference noted bold, italics and parentheses. N of at least 5 for each time point. Data represents a longitudinal study of the same animals throughout a 12 month period. Some animals were sacrificed at each time point for histological analysis.

## Discussion

*FKRP* mutations are associated with perhaps the widest clinical manifestation from mild LGMD2I to CMD and WWS with limited life span. One of the most prominent clinical phenotypes is the defect in cardiac function. Reports indicate that a majority of LGMD2I patients are associated with cardiomyopathies, from reduced ventricular EF to severe cardiac failure, and the most common pathophysiology evidence is a dilated cardiomyopathy. However, the severity of the cardiac defect appears not to be directly related to the severity in skeletal muscle or the involvement of other vital organs such as brain and visual system. Boito *et al* and Francesco Muntoni’s group have reported that patients with congenital muscular dystrophy type 1C (MDC1C) at young age could be without cardiomyopathy or only show mild impaired left ventricular function whereas those with mild LGMD2I at old age (40 years or older) often show severe dilated cardiomyopathy and die from left ventricular failure [34]. No clinical data is available to directly link the expression of F-α-DG to pathological changes and cardiac function, and no mechanism has been proposed to explain the wide phenotypic variation.

The *P448Lneo-* mouse used in this study exhibits phenotypic characteristics similar to those of LGMD2I without CNS involvement. We have previously reported that the young dystrophic mice (2 month of age or younger) present no apparent dystrophic pathology in heart muscle despite the fact that there were no detectable levels of F-α-DG. The current study revealed that physiological changes in fact become detectable, with smaller left ventricle DL and SL at 6 weeks of age, by echocardiogram. This is consistent with reports of cardiac involvement in the LGMD2I patients. Most significant pathophysiological changes in *P448Lneo–*mouse heart is the increased myocardial thickness. A further examination of the heart morphology demonstrates a smaller heart in the *P448Lneo-* animals compared to *C57* beginning at about 6 months of age. However, it is clear that, while the epicardial area normalizes around 10 months of age, the increased myocardial thickness leads to a reduced endocardial area throughout the 12 months period (S2 Fig). This appears to help the diseased mouse to maintain its cardiac output to near normal at an early age. However, cardiac output is clearly reduced from 6 months onwards when compared to normal *C57* mice. This together with the significant reduction in both EDV and ESV, especially during the 6–10 months period, suggests that the mutant mice are unable to maintain the normal levels of cardiac output despite the increase in myocardial thickness. The cardiac output, EDV and ESV maintain similar levels from 6 months to 12 months with relatively limited deterioration in disease progression during this period. This is consistent with the observation that accumulation of extracellular matrix and mononuclear cell infiltration is limited during the same period. In contrast, a small but clear decline of stroke volume and cardiac output is observed in normal *C57* mice at 12 months of age. The reason for the stabilization of cardiac function in *P448Lneo-* mutant mice during this period is not clear. One possibility is that the reduced muscle strength, therefore the activity, reduces the stress on cardiac muscle. This again allows the hypertrophic heart to maintain its main functions up to 12 months. This could also be the reasons for the lack of dilated cardiomyopathy in the *P448L-* mutant mice by this age. The difference in body size and thus the pressure required for pumping blood to reach whole body between mice and human also likely plays an important role for the difference in severity of cardiomyopathy. Thus limited cardiac muscle hypertrophy can be sufficient to compensate for the functional defects in mice for a prolonged time, but similar levels of hypertrophy will be insufficient to avoid cardiac failure such as dilated cardiomyopathy in human.

Milder cardiomyopathy appears to be a general feature of mouse models of muscular dystrophy when compared to patients in clinic. Cardiac dysfunction is also one of the common manifestation of DMD. However, Fayssoil *et al* examined cardiac function in mdx mice using high-resolution Doppler echocardiography [35] and only demonstrate limited defects in pathophysiology of the animals aged 10 and 12 months. The mdx heart has significantly larger posterior wall thickness but no significant difference in ejection fraction. There was no difference in heart rate in both ages and therefore overall cardiac output was normal. However, significant dilated cardiomyopathy can be demonstrated in older mdx mice (21 months old) [36]. It is possible that more prominent defects such as dilated cardiomyopathy could emerge in later stage of the *FKRP* mutant mice. Nevertheless, the clear functional defects detected by echocardiography in the *P448L* mutant mice provide highly useful markers to assess experimental therapy on cardiac functions of the disease[34].

There are clear differences in pathological changes in the different type of muscles of the mutant mice. All limb skeletal muscles present severe degeneration (necrotic fibers) and regeneration (centranucleation) as predominant feature with relatively limited fibrosis within the life time we examined. The cardiac muscle only presents very limited increase in fibrotic tissues. The most severe dystrophic phenotype of the *P448L* mutant mice occurs to the diaphragm, with clear loss of muscle fibers and decrease in size beginning as early as 6 weeks. The degeneration, inflammatory infiltration and fibrosis progress at higher speed than those in any other muscle tissues. By 6 months, nearly 50% of muscle mass has been replaced by collagen-rich ECM and other non-muscle components. Majority of muscle mass is lost by the end of 1 year. The severe dystrophic phenotype in the *P448L* mutant mice clearly indicates the importance of the glycosylation of α-DG in maintaining muscle integrity of the diaphragm. This is consistent with earlier reports from clinic that large proportion of LGMD2I patients (between 27 and 36% of compound heterozygotes and between 11 and 17% of homozygous) exhibit a loss of respiratory function at various ages and disease states requiring respiratory assistance [26,31]. However, it is not understood how the loss of F-α-DG affects diaphragm more than other skeletal and cardiac muscles. Clearly, loss of respiratory function contributes significantly to the mortality of the *P448Lneo-* mutant mice and likely to *FKRP*-related patients [25,34]. The marked pathology in the diaphragm and its effect on respiratory function also provide a highly valuable and convenient means of assessing experimental therapies to the diseases.

In summary, our study demonstrates that the *FKRP* mutant mice share pathophysiological features of human LGMD2I with disease progression in both skeletal and cardiac muscles, especially in diaphragm. The age related functional characteristics in cardiac muscle provide valuable markers for experimental therapy aiming to rescue the cardiac dysfunction of the diseases.

## Supporting Information

S1 Fig Western blot of *P448Lneo-* mutant and *C57/Bl6* normal control diaphragm and heart. Diaphragm and heart total proteins were incubated with IIH6C4 antibody for the presence of glycosylated α-DG. Control samples show a high expression of glycosylated a-DG in muscle fibers throughout a 12 month period. *P448Lneo-* diaphragm and heart show a lack of IIH6 expression in muscle fibers at 6 weeks of age and continuing through 12 months. Representative of 3 samples.
(TIF)

S2 Fig Elliptical representation of left ventricle (LV) of *P448Lneo-* and *C57* mice at 6–8 weeks, 6 months, 8 months, 10 months and 12 months. Epicardial and Endocardial shape were determined using endocardial and epicardial length as well as myocardial thickness values to determine elliptical shape. Column A shows the elliptical representation of the left ventricle shape comparing *C57* to *P448Lneo-* mice from 6 weeks of age to 12 months. Colum B shows an overlay of *C57* and *P448Lneo-* epicardium and endocardium shape from 6 weeks to 12 months of age. The *P448L* mutant mice appear to have smaller heart with smaller ventricle size and thicker cardiac walls. However, the epicardial length normalizes towards *C57* from around 10 months of age.
(TIF)

## References

[1] Ervasti JM, Ohlendieck K, Kahl SD, Gaver MG, Campbell KP (1990) Deficiency of a glycoprotein component of the dystrophin complex in dystrophic muscle. Nature 345: 315–319. 10.1038/345315a0 2188135

[2] Yoshida M, Ozawa E (1990) Glycoprotein complex anchoring dystrophin to sarcolemma. J Biochem 108: 748–752. 2081733 10.1093/oxfordjournals.jbchem.a123276

[3] Ervasti JM, Campbell KP (1991) Membrane organization of the dystrophin-glycoprotein complex. Cell 66: 1121–1131. 10.1016/0092-8674(91)90035-w 1913804

[4] Ervasti JM, Campbell KP (1993) A role for the dystrophin-glycoprotein complex as a transmembrane linker between laminin and actin. J Cell Biol 122: 809–823. 10.1083/jcb.122.4.809 8349731 PMC2119587

[5] Kanagawa M, Toda T (2006) The genetic and molecular basis of muscular dystrophy: roles of cell-matrix linkage in the pathogenesis. J Hum Genet 51: 915–926. 10.1007/s10038-006-0056-7 16969582

[6] Toda T, Kobayashi K, Kondo-Iida E, Sasaki J, Nakamura Y (2000) The Fukuyama congenital muscular dystrophy story. Neuromuscul Disord 10: 153–159. 10.1016/s0960-8966(99)00109-1 10734260

[7] Yoshida A, Kobayashi K, Manya H, Taniguchi K, Kano H, et al (2001) Muscular dystrophy and neuronal migration disorder caused by mutations in a glycosyltransferase, POMGnT1. Dev Cell 1: 717–724. 10.1016/s1534-5807(01)00070-3 11709191

[8] van Reeuwijk J, Janssen M, van den Elzen C, Beltran-Valero de Bernabe D, Sabatelli P, et al (2005) POMT2 mutations cause alpha-dystroglycan hypoglycosylation and Walker-Warburg syndrome. J Med Genet 42: 907–912. 10.1136/jmg.2005.031963 15894594 PMC1735967

[9] Roscioli T, Kamsteeg EJ, Buysse K, Maystadt I, van Reeuwijk J, et al (2012) Mutations in ISPD cause Walker-Warburg syndrome and defective glycosylation of alpha-dystroglycan. Nat Genet 44: 581–585. 10.1038/ng.2253 22522421 PMC3378661

[10] Ogawa M, Nakamura N, Nakayama Y, Kurosaka A, Manya H, et al (2013) GTDC2 modifies O-mannosylated alpha-dystroglycan in the endoplasmic reticulum to generate N-acetyl glucosamine epitopes reactive with CTD110.6 antibody. Biochem Biophys Res Commun. 10.1016/j.bbrc.2013.09.022 24041696

[11] Stevens E, Carss KJ, Cirak S, Foley AR, Torelli S, et al (2013) Mutations in B3GALNT2 cause congenital muscular dystrophy and hypoglycosylation of alpha-dystroglycan. Am J Hum Genet 92: 354–365. 10.1016/j.ajhg.2013.01.016 23453667 PMC3591840

[12] Vuillaumier-Barrot S, Bouchet-Seraphin C, Chelbi M, Devisme L, Quentin S, et al (2012) Identification of mutations in TMEM5 and ISPD as a cause of severe cobblestone lissencephaly. Am J Hum Genet 91: 1135–1143. 10.1016/j.ajhg.2012.10.009 23217329 PMC3516603

[13] Manzini MC, Tambunan DE, Hill RS, Yu TW, Maynard TM, et al (2012) Exome sequencing and functional validation in zebrafish identify GTDC2 mutations as a cause of Walker-Warburg syndrome. Am J Hum Genet 91: 541–547. 10.1016/j.ajhg.2012.07.009 22958903 PMC3512000

[14] Willer T, Lee H, Lommel M, Yoshida-Moriguchi T, de Bernabe DB, et al (2012) ISPD loss-of-function mutations disrupt dystroglycan O-mannosylation and cause Walker-Warburg syndrome. Nat Genet 44: 575–580. 10.1038/ng.2252 22522420 PMC3371168

[15] Buysse K, Riemersma M, Powell G, van Reeuwijk J, Chitayat D, et al (2013) Missense mutations in beta-1,3-N-acetylglucosaminyltransferase 1 (B3GNT1) cause Walker-Warburg syndrome. Hum Mol Genet 22: 1746–1754. 10.1093/hmg/ddt021 23359570 PMC3613162

[16] Jae LT, Raaben M, Riemersma M, van Beusekom E, Blomen VA, et al (2013) Deciphering the glycosylome of dystroglycanopathies using haploid screens for lassa virus entry. Science 340: 479–483. 10.1126/science.1233675 23519211 PMC3919138

[17] Stevens E, Carss KJ, Cirak S, Foley AR, Torelli S, et al (2013) Mutations in B3GALNT2 cause congenital muscular dystrophy and hypoglycosylation of alpha-dystroglycan. Am J Hum Genet 92: 354–365. 10.1016/j.ajhg.2013.01.016 23453667 PMC3591840

[18] Manya H, Chiba A, Yoshida A, Wang X, Chiba Y, et al (2004) Demonstration of mammalian protein O-mannosyltransferase activity: coexpression of POMT1 and POMT2 required for enzymatic activity. Proc Natl Acad Sci U S A 101: 500–505. 10.1073/pnas.0307228101 14699049 PMC327176

[19] Manya H, Sakai K, Kobayashi K, Taniguchi K, Kawakita M, et al (2003) Loss-of-function of an N-acetylglucosaminyltransferase, POMGnT1, in muscle-eye-brain disease. Biochem Biophys Res Commun 306: 93–97. 10.1016/s0006-291x(03)00924-0 12788071

[20] Goddeeris MM, Wu B, Venzke D, Yoshida-Moriguchi T, Saito F, et al (2013) LARGE glycans on dystroglycan function as a tunable matrix scaffold to prevent dystrophy. Nature 503: 136–140. 10.1038/nature12605 24132234 PMC3891507

[21] Ashikov A, Buettner FF, Tiemann B, Gerardy-Schahn R, Bakker H (2013) LARGE2 generates the same xylose- and glucuronic acid-containing glycan structures as LARGE. Glycobiology 23: 303–309. 10.1093/glycob/cws153 23135544

[22] Zhang P, Hu H (2012) Differential glycosylation of alpha-dystroglycan and proteins other than alpha-dystroglycan by like-glycosyltransferase. Glycobiology 22: 235–247. 10.1093/glycob/cwr131 21930648 PMC3255506

[23] Inamori K, Yoshida-Moriguchi T, Hara Y, Anderson ME, Yu L, et al (2012) Dystroglycan function requires xylosyl- and glucuronyltransferase activities of LARGE. Science 335: 93–96. 10.1126/science.1214115 22223806 PMC3702376

[24] Kanagawa M, Kobayashi K, Tajiri M, Manya H, Kuga A, et al (2016) Identification of a Post-translational Modification with Ribitol-Phosphate and Its Defect in Muscular Dystrophy. Cell Rep 14: 2209–2223. 10.1016/j.celrep.2016.02.017 26923585

[25] Stensland E, Lindal S, Jonsrud C, Torbergsen T, Bindoff LA, et al (2011) Prevalence, mutation spectrum and phenotypic variability in Norwegian patients with Limb Girdle Muscular Dystrophy 2I. Neuromuscul Disord 21: 41–46. 10.1016/j.nmd.2010.08.008 20961759

[26] Sveen ML, Schwartz M, Vissing J (2006) High prevalence and phenotype-genotype correlations of limb girdle muscular dystrophy type 2I in Denmark. Ann Neurol 59: 808–815. 10.1002/ana.20824 16634037

[27] Rasmussen M, Scheie D, Breivik N, Mork M, Lindal S (2014) Clinical and muscle biopsy findings in Norwegian paediatric patients with limb girdle muscular dystrophy 2I. Acta Paediatr 103: 553–558. 10.1111/apa.12561 24447024

[28] Pane M, Messina S, Vasco G, Foley AR, Morandi L, et al (2012) Respiratory and cardiac function in congenital muscular dystrophies with alpha dystroglycan deficiency. Neuromuscul Disord 22: 685–689. 10.1016/j.nmd.2012.05.006 22727687 PMC3476532

[29] Liang WC, Hayashi YK, Ogawa M, Wang CH, Huang WT, et al (2013) Limb-girdle muscular dystrophy type 2I is not rare in Taiwan. Neuromuscul Disord 23: 675–681. 10.1016/j.nmd.2013.05.010 23800702

[30] Wahbi K, Meune C, Hamouda el H, Stojkovic T, Laforet P, et al (2008) Cardiac assessment of limb-girdle muscular dystrophy 2I patients: an echography, Holter ECG and magnetic resonance imaging study. Neuromuscul Disord 18: 650–655. 10.1016/j.nmd.2008.06.365 18639457

[31] Poppe M, Bourke J, Eagle M, Frosk P, Wrogemann K, et al (2004) Cardiac and respiratory failure in limb-girdle muscular dystrophy 2I. Ann Neurol 56: 738–741. 10.1002/ana.20283 15505776

[32] Chan YM, Keramaris-Vrantsis E, Lidov HG, Norton JH, Zinchenko N, et al (2010) Fukutin-related protein is essential for mouse muscle, brain and eye development and mutation recapitulates the wide clinical spectrums of dystroglycanopathies. Hum Mol Genet 19: 3995–4006. 10.1093/hmg/ddq314 20675713

[33] Blaeser A, Keramaris E, Chan YM, Sparks S, Cowley D, et al (2013) Mouse models of fukutin-related protein mutations show a wide range of disease phenotypes. Hum Genet 132: 923–934. 10.1007/s00439-013-1302-7 23591631

[34] Boito CA, Melacini P, Vianello A, Prandini P, Gavassini BF, et al (2005) Clinical and molecular characterization of patients with limb-girdle muscular dystrophy type 2I. Arch Neurol 62: 1894–1899. 10.1001/archneur.62.12.1894 16344347

[35] Fayssoil A, Renault G, Guerchet N, Marchiol-Fournigault C, Fougerousse F, et al (2013) Cardiac characterization of mdx mice using high-resolution doppler echocardiography. J Ultrasound Med 32: 757–761. 10.7863/ultra.32.5.757 23620316

[36] Bostick B, Yue Y, Long C, Duan D (2008) Prevention of dystrophin-deficient cardiomyopathy in twenty-one-month-old carrier mice by mosaic dystrophin expression or complementary dystrophin/utrophin expression. Circ Res 102: 121–130. 10.1161/CIRCRESAHA.107.162982 17967782

