# Supplementary figures and images for: Progressive Dystrophic Pathology in Diaphragm and Impairment of Cardiac Function in FKRP P448L Mutant Mice
Source: PLoS One. 2016 Oct 6;11(10):e0164187. doi: 10.1371/journal.pone.0164187 (PMC5053477; doi:10.1371/journal.pone.0164187)

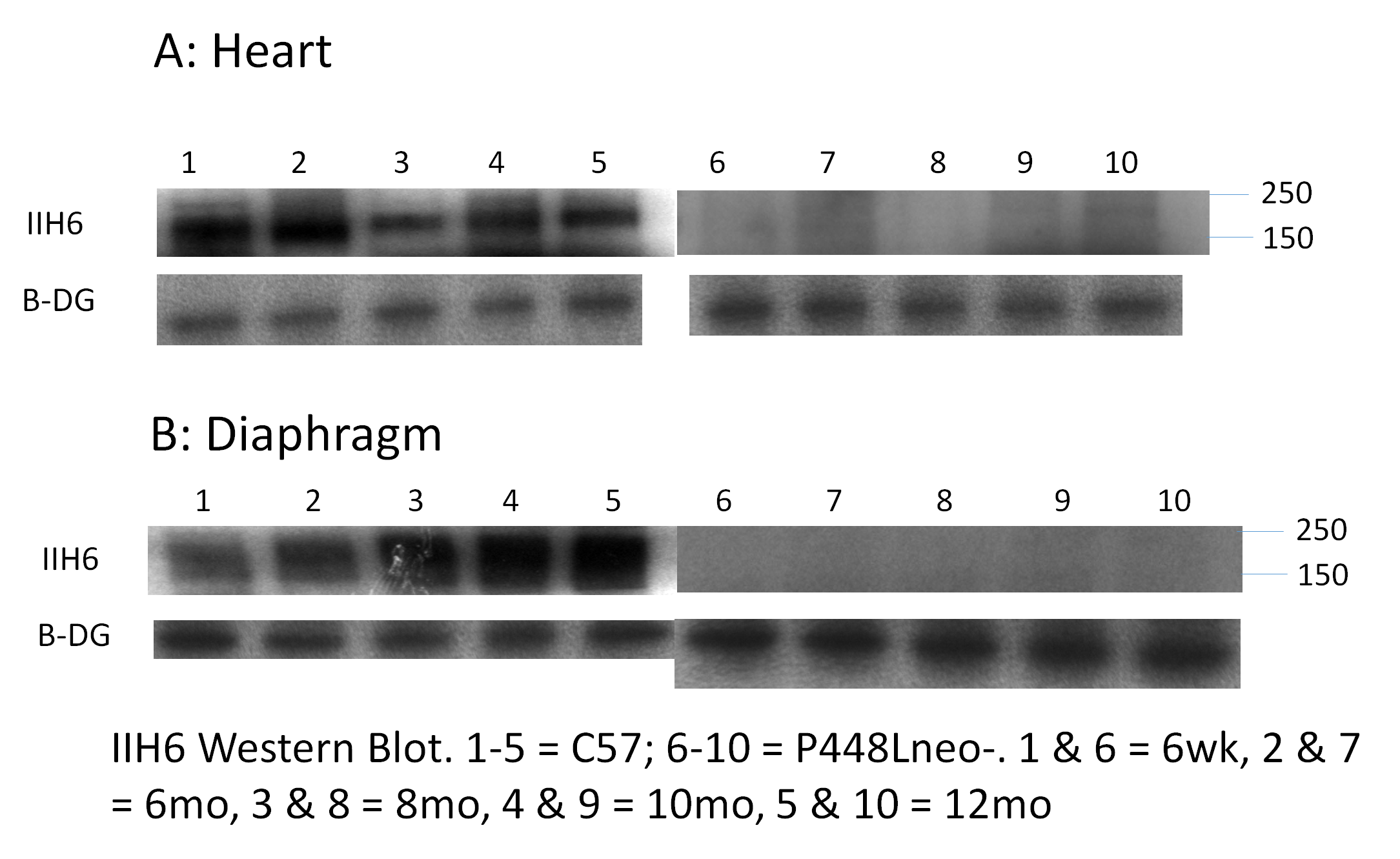

Supplement: S1 Fig — Diaphragm and heart total proteins were incubated with IIH6C4 antibody for the presence of glycosylated α-DG. Control samples show a high expression of glycosylated a-DG in muscle fibers throughout a 12 month period. P448Lneo- diaphragm and heart show a lack of IIH6 expression in muscle fibers at 6 weeks of age and continuing through 12 months. Representative of 3 samples. (TIF) [file pone.0164187.s001.tif]

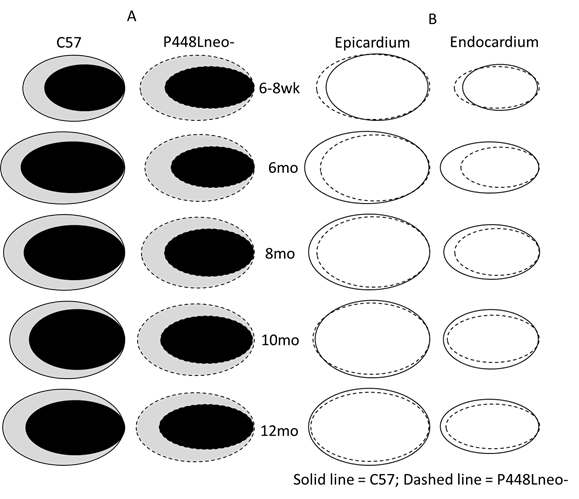

Supplement: S2 Fig — Epicardial and Endocardial shape were determined using endocardial and epicardial length as well as myocardial thickness values to determine elliptical shape. Column A shows the elliptical representation of the left ventricle shape comparing C57 to P448Lneo- mice from 6 weeks of age to 12 months. Colum B shows an overlay of C57 and P448Lneo- epicardium and endocardium shape from 6 weeks to 12 months of age. The P448L mutant mice appear to have smaller heart with smaller ventricle size and thicker cardiac walls. However, the epicardial length normalizes towards C57 from around 10 months of age. (TIF) [file pone.0164187.s002.tif]
